# Investigation of Structural and Spectral Peculiarities of *Fusarium* sp. Indicator Pigment Bostrycoidin

**DOI:** 10.3390/molecules29194765

**Published:** 2024-10-08

**Authors:** Anastasia Povolotckaia, Dmitrii Pankin, Vasiliy Novikov, Evgenii Borisov, Sergey Kuznetsov, Alexey Dorokhov, Anatoly Gulyaev, Elena Zavyalova, Rugiya Alieva, Sergey Akulov, Sergey Belousov, Maksim Moskovskiy

**Affiliations:** 1 Federal Scientific Agro-Engineering Center VIM, 1st Institutskiy Proezd 5, 109428 Moscow, Russia; vasiliy1992@gmail.com (V.N.); kuznetsov.sm.93@gmail.com (S.K.); dorokhov@rgau-msha.ru (A.D.); zlenka2006@gmail.com (E.Z.); serg.akulov@gmail.com (S.A.); maxmoskovsky74@yandex.ru (M.M.); 2 Center for Optical and Laser Materials Research, St. Petersburg State University, Ulianovskaya 5, 198504 St. Petersburg, Russia; dmitrii.pankin@spbu.ru (D.P.); eugene.borisov@spbu.ru (E.B.); 3 Chemistry Department, Lomonosov Moscow State University, 119991 Moscow, Russia; ruqiwa_eva@mail.ru; 4 Department of Processes and Machines in Agribusiness, Kuban State Agrarian University Named after I.T. Trubilin, 350044 Krasnodar, Russia; sergey_belousov_87@mail.ru

**Keywords:** bostrycoidin, DFT, IR absorbance, vibrational spectroscopy, UV–Vis absorbance

## Abstract

Bostrycoidin is one of the pigments produced by the *Fusarium* genus of fungi. On the one hand, it has significant pharmacological importance, while on the other hand, it serves as a presence marker of Fusarium infection in useful grain crops, fruits, and soils. In this regard, the structural and optical properties of the bostrycoidin molecule were studied in the framework of density functional theory (DFT). The most stable geometry as well as higher-energy conformers and tautomers were investigated. The lowest-energy tautomer was found to be about 3 kcal/mol higher in energy than the most stable structure, resulting in relatively low population of this state. The obtained conformational rotamers associated with the rotation of the OMe group possess similar energy. The vibrational spectrum was modeled for the most stable conformer, and the most active peaks in the IR absorbance spectrum were assigned. Moreover, the electronic absorption spectrum was simulated within the time-dependent DFT approach. The obtained theoretical spectrum is in good agreement with the experimental data and the theoretically calculated longest-wavelength transition (HOMO–LUMO) was about 498 nm.

## 1. Introduction

Bostrycoidin (CAS 4589-33-7, 5,8-Dihydroxy-6-methoxy-3-methyl-2-aza-9,10-anthraquinone or 6,9-Dihydroxy-7-methoxy-3-methylbenz[g] isoquinoline-5,10-dione [1] is one of the pigments produced by the *Fusarium* genus of fungi. Its isolation from *F. Bostrycoides* was first reported in a 1954 paper by Cajori et al [2,3]. To date, bostrycoidin and its derivatives have been isolated from fungi such as *Fusarium oxysporum* [4,5], *Fusarium solani* [6,7,8,9,10] and *Artonia Cinnabarina* [11].

From the very first studies, the high biological activity of this pigment was noted, including antibacterial properties and effectiveness against tuberculosis bacillus, strain H37, in vitro [2]. Further studies demonstrated the activity of the pigment against gram-positive bacteria as well as yeast [1]. In this regard, studying the pathways for the synthesis of this substance is relevant from both fundamental and applied points of view. Fundamental interest is associated with understanding the genes, conditions, and mechanisms of reactions [12] that promote the biosynthesis of this substance in the fungus. The applied interest is connected with the synthesis of this compound for the abovementioned pharmacological needs, as well as the large-scale synthesis of eco-friendly pigments for the textile industry [8]. Since the pigment bostrycoidin is typical for the *Fusarium* genus of fungi [2,4,5,6,7,8,9,10], which causes Fusarium wilt in cereal crops and nightshades that are important for food security, an equally important practical task is the identification of grains, fruits, and soil contamination [13,14]. In this regard, the development of methods for identifying metabolites, including pigments like bostrycoidin, is practically important. In turn, this requires fundamental research into the properties closely related to the structure of bostrycoidin.

Currently, the most commonly used methods for identifying this pigment and its derivatives are chromatography [15,16], mass spectrometry [11], NMR [7,9,11,15,17,18], vibrational spectroscopy (mainly IR absorption) [2,9,11,18], and UV–Vis absorption spectroscopy [2,19].

The optical spectroscopy methods—vibrational (Raman and IR absorption spectroscopy) and UV–Vis absorption spectroscopy—are very promising for streaming applications for identifying substances among all of the listed methods [20,21,22]. Optical methods are relatively simple to implement and possess high sensitivity to the structure of the studied substance. In addition, the use of chemometric analysis methods and machine learning algorithms can improve the accuracy of identification and increase the scale of testing [22,23,24,25,26]. This is especially important during the food product contamination analysis as well as when monitoring the condition of soils and grain materials stored in warehouses.

Another advantage of optical spectroscopy methods is the possibility of theoretical calculation of optical properties using fairly accurate quantum chemical methods, which allows reliable prediction of properties, especially important in the case of complex biological systems. For example, using quantum chemical methods within the framework of density functional theory, optical properties were theoretically calculated and compared with available experimental data for a number of pigments typical for the *Fusarium* genus of fungi, such as various carotenoids [27,28,29], rubrofusarin [30,31], anhydrofusarubin [32]. The latter pigment is structurally close to bostrycoidin, although it has a number of specific biologically active properties [33,34]. This additionally motivates this study of the structure–spectrum correlation in order to find common spectral features, as well as those that can be used for differentiation.

Due to the lack of any quantum chemical studies of the bostrycoidin molecule, as well as limited experimental data on its structure and optical properties, this work aims to study the structure of the bostrycoidin molecule in the ground state, possible tautomers and conformational isomers, as well as vibrational properties and electronic spectrum in the practically important UV–Vis range. The obtained results are discussed together with the available experimental results for bostrycoidin, as well as experimental and theoretical results for the structurally related anhydrofusarubin.

## 2. Theoretical Approach

The quantum chemical study of the bostrycoidin molecule was performed within the framework of the density functional theory (DFT) using the B3LYP exchange-correlation functional [35,36,37] with a split-valence triple-zeta Pople’s basis set 6-311G(2d,p) [38,39]. Previously, such approach had demonstrated good agreement between theoretically predicted and experimentally observed structural and optical properties [31,32,40]. At the initial stage, the potential energy was scanned with a step of 15° with the change in the dihedral angle. Additionally, the potential energy scans with 0.07 Å step bond length change were obtained. Further, the obtained approximate geometries of minima and transition states were refined. The procedure was performed until the standard criteria of smallness of the maximum and root-mean-square forces and atomic displacements were met. The absence of imaginary frequencies was checked for the most stable state and higher-energy minima (higher-energy stable states). The transition states were optimized with one imaginary frequency. In order to compare, the energies of minima and potential energy barriers were estimated for the cases of the molecule in the gas phase (GP, the case of non-interacting molecule) and in chloroform (CHCl_3_) solution, implicitly taking into account the solvent effect with the polarizable continuum model (PCM) [41,42,43] and standard parameters. The theoretical vibrational spectrum was calculated for the molecule in GP for greater generality and also in CHCl_3_ solution. In the case of GP, the scaling procedure was performed in order to take into account the condensation effects on mode frequencies. For better agreement with the experimental vibrational spectrum (Raman and IR absorbance spectra), the text discusses scaled frequencies of vibrational modes (scaling factor 0.98, taken from [32]). Vibrational modes interpretation is based both on the potential energy distribution obtained with Veda 4 software [44,45] and on the calculated atomic displacements. For resulting IR absorbance spectra, a 4 cm^−1^ half-width at half-maximum of peak was set. The calculation of the UV–Vis absorbance spectrum was carried out in the approximation of singlet-singlet transitions for the bostrycoidin molecule in chloroform solution within the time-dependent DFT approach [46,47]. Forty lowest-energy electronic singlet-singlet transitions were calculated. This approach allowed obtaining data in the practically important region of 185–700 nm. The final contour of the absorption spectrum was formed with the Gaussian-type broadening of 0.33 eV. The calculations were performed using the Gaussian G09W Rev. C (Gaussian Inc., Wallingford, CT, USA) software [48], and visualization was performed using the GaussView 6.0.16 (Gaussian Inc., Wallingford, CT, USA) software.

## 3. Results and Discussions

### 3.1. Structural Peculiarities

#### 3.1.1. The Most Stable State

**Figure 1:**
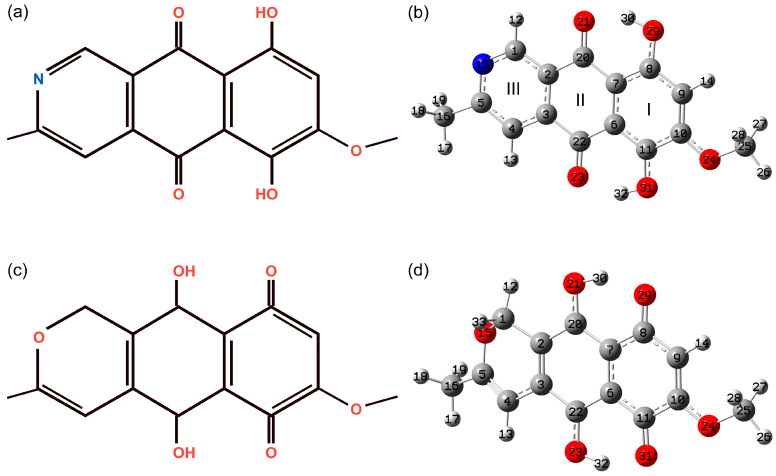
The schematic representation of bostrycoidin molecule (**a**) and corresponding optimized structure (**b**) as well as schematic representation of anhydrofusarubin molecule (**c**) and corresponding optimized structure (**d**). The I, II, and III are the ring numbers. Carbon, oxygen, nitrogen, and hydrogen atoms hereinafter were marked with dark gray, red, blue, and light gray colors.

The bostrycoidin molecule contains a rigid polycyclic skeleton. The optimized geometry of the bostrycoidin molecule is shown in Figure 1. The most stable structure in the gas phase (GP) and the chloroform solution corresponds to a molecule with the point group symmetry Cs, where the mirror plane coincides with the plane of the polycyclic skeleton. By analogy with anhydrofusarubin (Figure 1), the naphthazarin part can be distinguished in the polycyclic skeleton. However, unlike anhydrofusarubin, the naphthazarin part is located in a way, where the quinone ring is in the middle of the molecule’s polycyclic skeleton and the benzene ring is at the edge for the ground state. A comparison of the bond lengths and angles in rings I and II with those in the anhydrofusarubin molecule is presented in Table 1.

As can be seen for the ring I in bostrycoidin, due to the close location of the OH and OMe groups, an elongated value of the C10C11 bond is noted with a simultaneous shortening of the C9C10 and C11C6 bonds. In the case of anhydrofusarubin, the OMe group is attached to the quinone ring and leads to an elongation of the C10C11 bond compared to a similar one in bostrycoidin (C22C3). At the same time, in naphthazarin, the longest bonds are ones analogous to C8C9 and C10C11 in bostrycoidin (Table S1). Due to the C_2v_ point group symmetry of the molecule [49], the lengths of these bonds in naphthazarin are equal, while in bostrycoidin, due to the presence of the MeO group, the length of the C10C11 bond is greater than C8C9. The shortest carbon bond in the ring I of bostrycoidin is C9C10. A similar result is observed in anhydrofusarubin and naphthazarin. The fairly long bonds in ring II are C2C20 and C22C3. In this case, the double bonds C=O are shifted in the direction of the OH groups, with which hydrogen bonds are formed. Thus, the distance between the atoms O21 and O29, as well as O23 and O31, is approximately equal to 2.55 Å. At the same time, the length of the hydrogen bond O21---H30 (1.642 Å) is shorter than O23---H32 (1.651 Å). Comparison with similar bonds in naphthazarin (O---H length 1.683 Å) shows the presence of a stronger hydrogen bond in bostrycoidin (see Table S1). For the third ring, the shortest bonds are C5N15 (1.346 Å) and N15C1 (1.326 Å). Carbon-carbon bonds have lengths in the range of 1.39–1.398 Å. In general, the ratios of the corresponding bond lengths in the analogous naphthazarin parts of the molecules of bostrycoidin, anhydrofusarubin, and naphthazarin are carried out, taking into account their symmetry features. The difference in the analogous plane angles, with the exception of C11C10O24 for bostrycoidin and anhydrofusarubin, as well as for bostrycoidin and naphthazarin, is within 1° (Table 1 and Table S1).

#### 3.1.2. Conformational Enantiomers of Bostrycoidin

The properties of biological molecules are closely related to both their structure and the properties of the environment, e.g., solution. The aforementioned chloroform is a solution widely used in biological applications. The next step consisted of the evaluation of higher-energy states, in particular conformational isomers and tautomers. 

Since the most flexible bond subjected to rotation in the bostrycoidin molecule is the CO bond, through which the OMe group is attached to the polycyclic skeleton, first of all, a scan was performed along the angle C11C10O24C25 (see the notation in Figure 1). The global minimum corresponds to the value 180° of C11C10O24C25. At the same time, local minima were noted at the C11C10O24C25 angle values of −41° and 41° and the C9C10O24C25 angle values of 145.3° and −145.3° for conformational enantiomers, correspondingly, the closest contact CH---O is 2.414 Å. An angle value close to 150° analogous to the C9C10O24C25 angle in anhydrofusarubin was obtained [32]. However, the closest contact was longer and had a value of 2.454 Å. 

**Figure 2:**
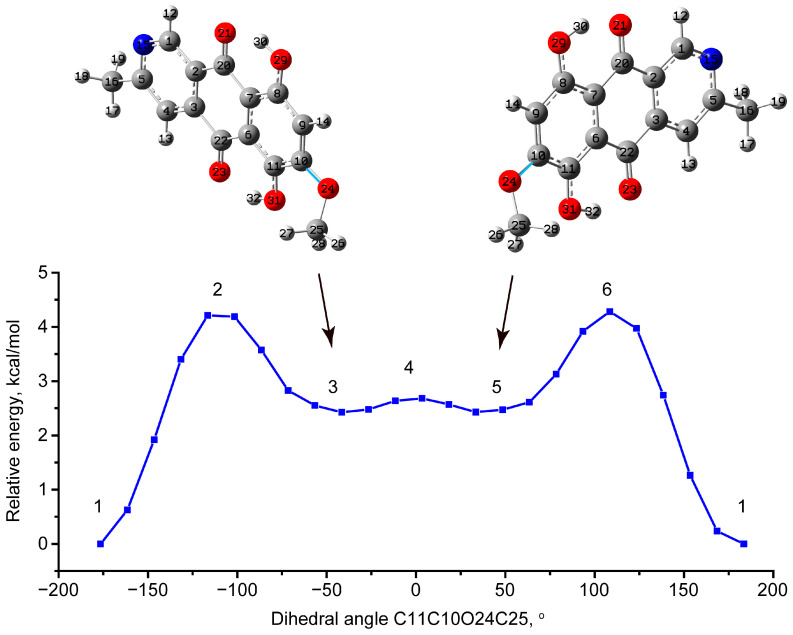
Potential energy scan with C11C10O24C25 dihedral angle change for the molecule in gas phase. The C10O24 bond is highlighted with cyan color. The optimized structure of stable conformational isomers 3 and 5 is demonstrated. The geometries of transition states 2, 4, and 6 are demonstrated in Table S1.

Considering bostrycoidin, the transition from the most stable state to the higher-lying conformational isomer involves overcoming a potential barrier of about 4.29 and 4.73 kcal/mol in GP and chloroform solution (Figure 2 and Figure S1). The local minima in solution have a higher relative energy (3.54 kcal/mol) than those in the gas phase (2.41 kcal/mol). In both cases, the potential barrier to the transition from one conformational enantiomer to another is very low (about 0.3 kcal/mol) (Figure S1 and Table S2). A comparison of the GP calculation results for bostrycoidin and anhydrofusarubin [32] yields comparable high energies of the conformational isomers and potential barriers to the rotation of the MeO group. Due to the equal energy of states 3 and 5 (degeneracy of energy level is 2), the probability, using the Boltzmann distribution, was estimated as 2e^−ΔE/kT^ (where ΔE is about 4.29 and 4.73 kcal/mol in GP and chloroform solution, and kT is about 0.6 kcal/mol). Such relatively high energies make states 3 and 5 only about 0.1% populated in total as compared to the most stable state at room temperature.

#### 3.1.3. Tautomeric Forms of Bostrycoidin

Both naphthazarin and anhydrofusarubin [32,50] have a significant proportion of tautomers formed as a result of hydrogen transition from the OH group to oxygen at O=C already at room temperature. 

**Figure 3:**
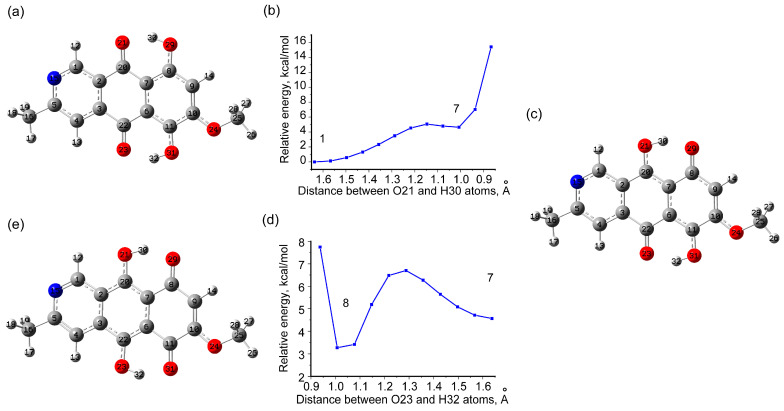
Path 1: scans of potential energy during the transition of one hydrogen from the OH group to oxygen in O=C, from the ground state (**a**) through the distance change between O21 and H30 atoms (**b**) to the intermediate minimum 7 (**c**), and from intermediate minimum 7 through the distance change between O23 and H32 (**d**) to the intermediate minimum 8 (**e**). The data about exact energies and transition states is listed in Table S3.

**Figure 4:**
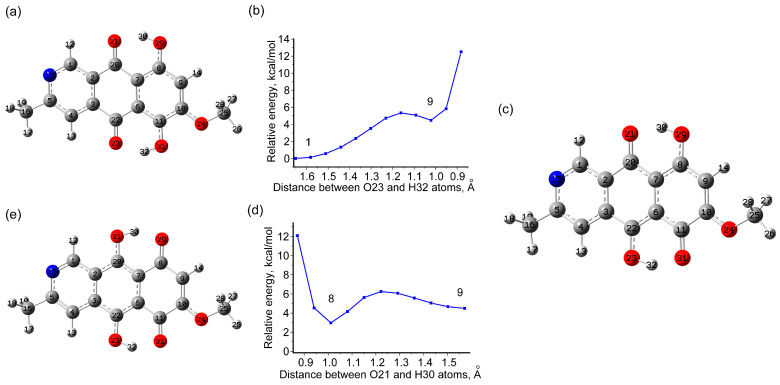
Path 2: Scans of potential energy during the transition of one hydrogen from the OH group to oxygen in O=C, from the ground state (**a**) through the distance change between the O23 and H32 atoms (**b**) to the intermediate minimum 9 (**c**), and from intermediate minimum 9 through the distance change between O21 and H30 (**d**) to the intermediate minimum 8 (**e**). The data about exact energies and transition states are listed in Table S3.

In this regard, the potential barriers and energy differences for the transition to various tautomeric states for the bostrycoidin molecule were estimated. The obtained results are presented in Figure 3 and Figure 4 and Table S3. Two pathways for successive transfer of 1 hydrogen at a time, so that OH groups are initially formed on either side of the C7C6 bond and then the stable state 8 is obtained (Figure 3 and Figure 4), could be considered. In both cases, the first step involves a transition to a state (states 7 in Figure 3 and states 9 in Figure 4) with a relatively high potential barrier to the transition from the ground state of 5.08 (4.93) kcal/mol for the 1→7 transition in GP (chloroform) and 5.38 (5.13) kcal/mol for the 1→9 transition in GP (chloroform). In this case, states 7 and 9 are higher in energy than the ground state by 4.57 (4.44) and 4.48 (4.16) kcal/mol in GP (chloroform). The further transition to state 8 is associated with overcoming an additional barrier of about 2 kcal/mol. As a result, the tautomeric state 8 turns out to be higher in energy by about 2.96 (2.83) kcal/mol than the most stable structure in GP (chloroform), making it extremely low populated at room temperature. For example, for a degenerate two-level system at 298.15K, the higher level with ΔE = +2.96 (2.83) kcal/mol has a population of about 1% (e^−ΔE/kT^) with respect to the most stable state.

Relative energy estimates show that under normal conditions the concentrations of conformational enantiomers 3 and 5, as well as tautomer 8, amount to a single percent. Hypothetically, a change in the ratio of the ground state to the abovementioned higher states may affect biological activity. However, the contribution of these higher states to the vibrational and electronic properties will be relatively small. Thus, for the bostrycoidin molecule, in contrast to anhydrofusarin and naphthazarin, an extremely insignificant number of tautomers and conformational isomers was predicted. Attempts to consider tautomeric transitions starting from higher intermediate states, such as intermediate states 3 and 5, lead to even higher-energy (less probable) states.

### 3.2. Optical Properties

#### Vibrational Spectroscopy

To the best of our knowledge, at the moment, only the article [2] provides the IR transmission spectrum of bostrycoidin. Due to the fact that the transmission value is given depending on the wavelength, in some cases, the wavelength in microns corresponding to the vibrational mode frequency is additionally given. A number of works provide several peaks identified in the IR absorption spectra as well. In our opinion, the most complete information is provided in the works [11,18]; the data from these works were taken to discuss the results obtained. 

**Figure 5:**
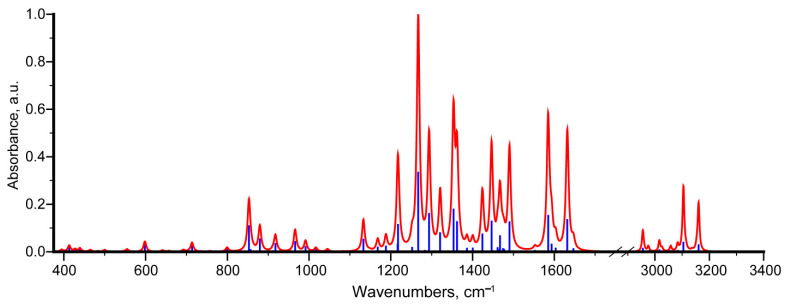
Scaled theoretical IR absorption spectrum of bostrycoidin molecule.

For greater practical relevance, the text mostly analyzes the case of a molecule in the GP in order not to be limited to a specific environment. The interpretation of vibrational modes in the practically important region 375–4000 cm^−1^ is given in the Supporting Material, especially in Table S4. For GP, the vibrational frequencies given here and below are scaled for greater practicality. Since IR absorption spectroscopy is used to identify this pigment, the vibrational spectrum analysis is given below with an emphasis on this method (Figure 5). In addition, the scaled Raman activity spectrum for possible future studies is given in the Supporting Materials (Figure S2).

According to the frequency value, the predicted vibrational modes using the harmonic approximation can be divided into two ranges: higher-frequency region (more than 2800 cm^−1^) and lower-frequency region (less than 1700 cm^−1^).

The hydrogen stretching vibrations of various origins are located in the high-frequency range. In this range, the most active modes in the IR spectrum are the stretching vibrations in the OH groups (modes no. 90 and 86), which is similar to the case of the anhydrofusarubin molecule [32]. Among these modes, mode no. 90 (v(H32O31)) has a higher-frequency, which correlates with the shorter H32O31 bond length compared to the H30O29 bond length, as well as the larger H32---O23 distance (1.651 Å) compared to H30---O21 (1.643). Hydrogen stretching vibrations at carbons with sp^3^ and sp^2^ hybridizations are much less intense. Calculations predict that the frequencies of vibrational modes with atomic displacements of hydrogens at carbons with sp^2^ hybridization (modes 87–89) are located between the frequencies of modes no. 90 and 86, occupying the range 3110–3155 cm^−1^. Hydrogen stretching vibrations at carbons with sp^3^ hybridization are localized in methyl groups. In this case, in descending order of frequency, one can distinguish antisymmetric methyl vibrations (modes no. 84 and 85), antisymmetric water-like vibrations (modes no. 83, 82), and symmetric methyl vibrations (modes no. 81, 80). It should be noted that calculations predict, as a rule, overestimated frequencies for hydrogen stretching vibrations, which is associated with a significant manifestation of anharmonicity of the X-H bond [40], as well as a significant influence on the environment. Thus, the typical experimental frequency range for vibrational modes with atomic displacements as in modes 87–89 is 3000–3100 cm^−1^, and for modes no. 84, 85, and no. 81, 80 is 2960 ± 10 cm^−1^ and 2870 ± 10 cm^−1^, correspondingly [51,52].

The most active peaks in the IR absorption spectrum are concentrated in the range of less than 1700 cm^−1^. In the calculated spectrum, one can distinguish the spectral range 1540–1665 cm^−1^ (approximately 6.5–6 μm), where modes no. 74–79 are located. A characteristic feature of these modes is a significant contribution from various vibrations in double bonds (C=C, C=O, C=N). The most intense modes in this range are modes 75 (1585 cm^−1^) and 78 (1632 cm^−1^).

Since mode no. 78 possesses a higher-frequency a mixed nature of atomic displacements is noted, including a larger amplitude v(C=O), coupled with v(C=C), v(C=N), and δ(COH). Moreover, in the case of the 3rd ring, the ratio of the amplitudes of atomic displacements resembles the analog in the v8b mode in the benzene ring. Here and thereafter, the Wilson notations [51] are used in order to briefly describe the carbon atom displacements. In turn, for mode no. 75, a larger amplitude of atomic displacements is predicted precisely in the third ring, i.e., the v(CC) and v(CN) vibrations have the largest amplitude, which correlates with the lower mode frequency. In the calculated structurally close spectrum of anhydrofusarubin, the presence of a mode with a frequency of 1590 cm^−1^ (mode no. 77) and two modes close in frequency and quite intense were predicted—1620 and 1631 cm^−1^ (modes no. 79 and 80) [32]. In the experimental IR transmission spectrum of bostrycoidin, presented in [2], the presence of two pronounced bands in this range is noted. In later works [11], as well as [18], the peak frequencies of 1590 and 1620 cm^−1^, as well as 1586 and 1623 cm^−1^, are clearly manifested. 

In the range of 1400–1500 cm^−1^ (7.1–6.7 μm) in the IR absorption spectrum, the presence of four fairly intense modes is predicted, its characteristic features are atomic displacements of the following types: bending hydrogen vibrations in methyl groups, stretching vibrations in double carbon bonds mainly in the benzene ring (ring 1), as well as bending vibrations with the participation of OH groups (δ(COH)). The most active modes in the spectrum are predicted to be modes no. 65 (1424 cm^−1^), 66 (1447 cm^−1^), 70 (1467 cm^−1^), and 73 (1490 cm^−1^). The IR transmission spectrum of bostrycoidin in [2] shows a complex-shaped transmission band in this range, consisting of several peaks. In this case, the transmission minima are achieved closer to the short-wavelength boundary and slightly less than 7 μm (approximately 1430 cm^−1^). At the same time, in [18], a peak at 1465 cm^−1^ is noted for bostrycoidin, and a pair of peaks is observed in naphthazarin near 1457 and 1409 cm^−1^ [53]. It should be noted that due to the significant contribution of bostrycoidin to the predicted vibrational modes in this range of hydrogen vibrations, a significant influence of the environment on the activity in the spectrum and, accordingly, on the observed position of the band maximum in the experiment is possible.

**Figure 6:**
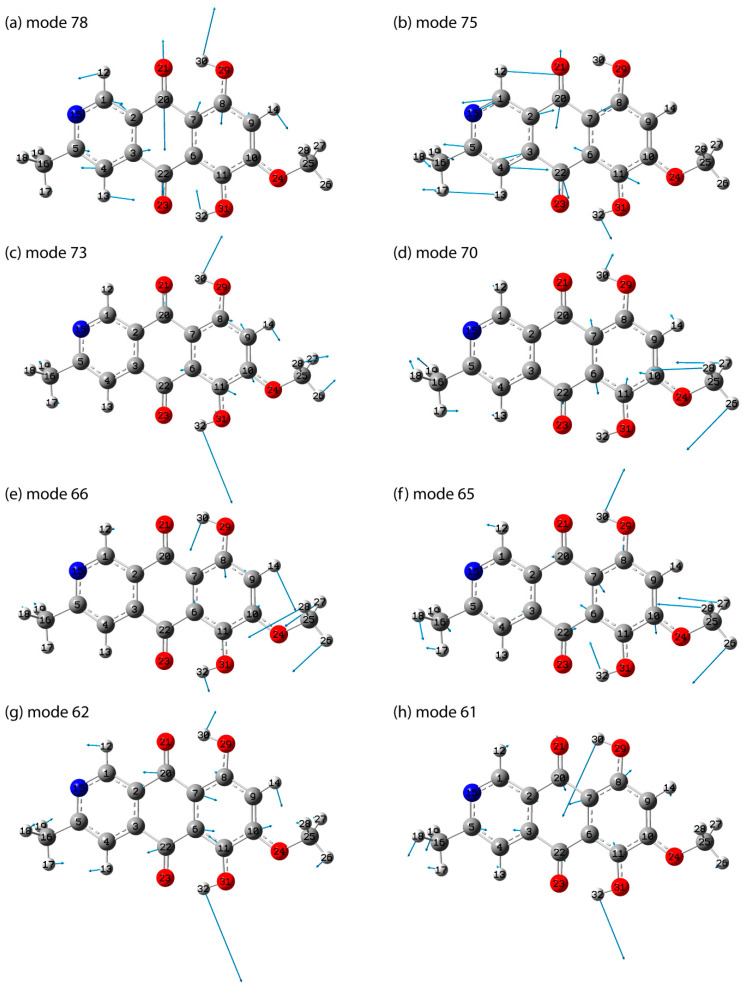
Atomic displacements in the selected vibrational modes: 78 (**a**), 75 (**b**), 73 (**c**), 70 (**d**), 66 (**e**), 65 (**f**), 62 (**g**), 61 (**h**).

Also, a significant contribution of hydrogen bending vibrations in COH is noted for modes no. 62 (1362 cm^−1^) and 61 (1353 cm^−1^), along with delocalized carbon vibrations in the rings (Figure 6).

The IR transmission spectrum [2] also shows a peak in this region, but its relative intensity is very low, which may be due to the influence of the environment. In [18], the peak frequency is 1383 cm^−1^, while in [11], this peak was not indicated. At the same time, a fairly significant localization of atomic displacements in the naphthazarin part suggests the presence of similar modes in approximately the same range. In the experimental IR spectrum from [53], the presence of a peak at 1343 cm^−1^ was noted.

The most active band in the experimental IR spectrum from [2] is just under 8 μm (1250 cm^−1^). In [11], a peak at 1260 cm^−1^ was noted in this range, while in [18], this maximum was near 1292 cm^−1^. The calculation predicts the presence of an intense mode no. 57 (1267 cm^−1^), as well as higher-frequency modes no. 59 (1294 cm^−1^) and 60 (1321 cm^−1^), their interpretation is given in Table S4. Presumably, the contribution of these modes leads to the short-wavelength shoulder observed in the IR transmission spectrum in [2]. For mode no. 57, a complex nature of atomic displacements is noted. In neighboring rings, the atomic displacements of carbons occurring in antiphase (and for ring 3 of nitrogen), resemble carbon displacements in mode v19b in the benzene ring. There is also a contribution from δ(N15C1H12), δ(C5C4H13), δ(C8O29H30), and v(C10O24) vibrations (Table S4). For mode no. 59, the predominant atomic displacements occur in v(C7C6), v(O31C11), v(C8CO29), v(O24C10), δ(C10C9H14), and δ(C8O29H30), with a smaller contribution from v(N15C1) and v(C5C4). As can be seen, for this mode, displacements occur in a number of polar bonds near the COH groups, which can be sensitive to the environment and lead to variable activity in the spectrum. This may explain the fact that different peak frequencies are given in different works [11,18].

In addition to the above, the experimental data from [18] note the presence of a peak at 1198 (≈8.35 μm) and 1136 cm^−1^ (≈8.8 μm). The latter is observed in [2]. The calculated spectrum shows the presence of a close in frequency and relatively intense vibrational modes no. 55 (1218 cm^−1^) and no. 50 (1133 cm^−1^), respectively. For mode no. 55, a contribution from v(O24C10) was noted, along with δ(C10C9H14), ρ(C25H3), and δ(O24C25H26), which made the mode quite polar and active in the spectrum. In mode no. 50, atomic displacements are much more strongly delocalized in the polycyclic skeleton. In the region from 800 to 1100 cm^−1^, the calculation predicts the most active modes in the IR absorption spectrum, no. 38 (853 cm^−1^) and no. 41 (880 cm^−1^)—their characteristic feature is significant atomic displacements of the hydrogen atom in CCOH. At the same time, for the modes that include a contribution from deformation vibrations in the polar groups CCO and CCN, for example, modes no. 43 (918 cm^−1^) and no. 44 (956 cm^−1^), the activity in the IR absorption spectrum is lower. In the region of 385–800 cm^−1^, there are two types of vibrational modes. The first type includes modes with atomic displacements in the plane of the rings. The second type includes out-of-plane vibrations (torsion modes). As the frequency decreases, the relative amplitude of the carbon, nitrogen, and oxygen atoms becomes increasingly larger. A qualitatively similar interpretation is noted in anhydrofusarubin. Additionally, the vibrational properties were calculated with implicit consideration of the solvent CHCl_3_ influence using PCM (see Figures S3 and S4, as well as Table S5). This approach allowed to correlate the corresponding vibrational modes in GP and solution based on atomic displacements and to estimate the changes in frequencies. The obtained result is presented in Figure S3 and Table S5. As can be seen, the most significant discrepancies are noted above 850 cm^−1^. Taking into account that the influence of the environment mostly leads to a decrease in the frequency, the frequency difference (frequency in gas phase minus frequency in CHCl_3_) becomes positive. Significant frequency differences mostly occur for vibrational modes where an important contribution is made by atomic displacements involving hydrogen and especially OH groups and polar bonds, especially C=O in 1615–1700 cm^−1^. Thus, a frequency difference of more than 10 cm^−1^ is noted for modes no. 38 and 41 (associated with torsional vibrations of OH groups), no. 59 (associated with deformation hydrogen vibrations), 78 (associated with stretching vibrations in C=O bonds and deformation hydrogen vibrations in OH groups), and no. 86 and 90 (associated with hydrogen stretching vibrations of OH groups).

### 3.3. UV–Vis Absorbance Spectroscopy

**Figure 7:**
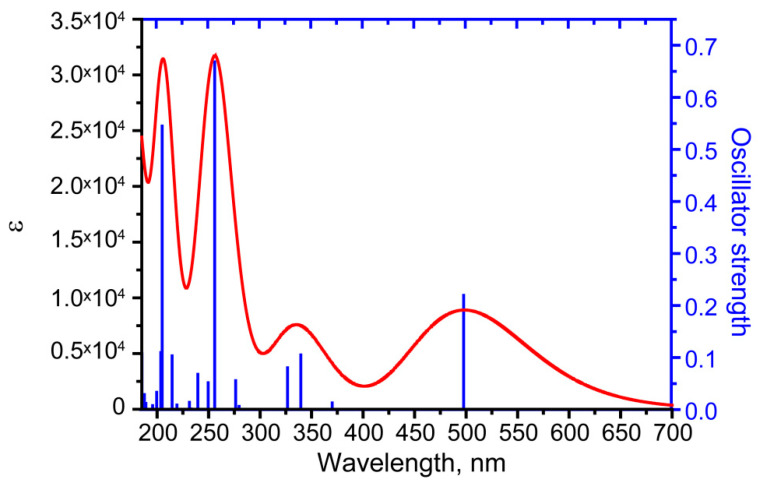
The UV–Vis spectrum in the 185–700 nm region for single bostrycoidin molecule in chloroform.

**Figure 8:**
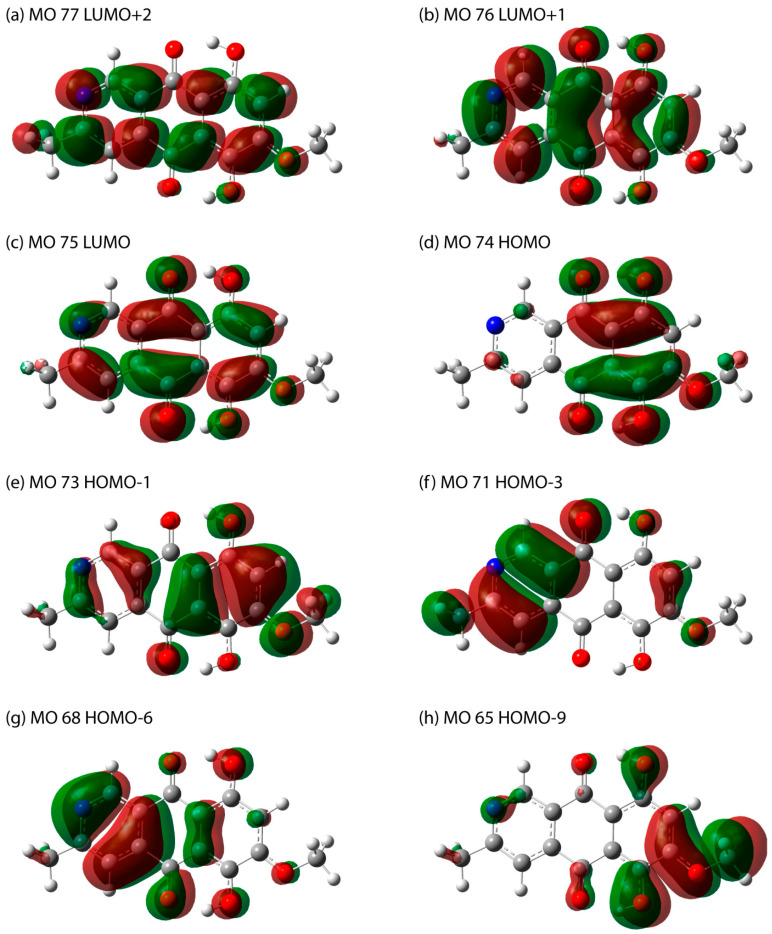
The molecular orbitals involved in singlet-singlet electron transitions with the highest oscillator strength are listed in Table 2.

In order to simulate the electronic absorption spectrum, a single molecule was optimized to the ground state in the practically important case of a chloroform solution, which effect was taken into account implicitly. In the singlet-singlet approximation, 40 lowest-energy transitions were calculated. The resulting spectrum with a broadening of 0.333 eV is shown in Figure 7. The energies (wavelengths) of the transitions, the participating molecular orbitals, their percentage contribution, and their appearance are presented in Table 2 and Figure 8. The calculated UV–Vis absorbance spectrum showed the presence of 4 bands, which coincides with the data given in [2,19]. The longest-wavelength experimental band corresponded with a fairly high accuracy to the theoretically predicted peak at about 500 nm, corresponding to the HOMO–LUMO transition. A long wavelength can be potentially useful for recording resonance Raman scattering, which emphasizes the need to study the vibrational properties of the substance as well. Additionally, due to the complex origin of bostrycoidin pigment synthesis and existence in nature, the UV–Vis spectra of tautomers were calculated. The Supplementary Materials provide comments, as well as the obtained UV–Vis absorption spectra in Figure S5. The wavelengths (energies) of the transitions that make the greatest contribution to the peak formation are presented in Table S6.

Considering bostrycoidin, the energy of this transition is higher than the energy of the analogous HOMO–LUMO transition in anhydrofusarubin (maximum in the range of 540–570 nm) [32], which is convenient electromagnetic region for absorption detection and resonant Raman spectra excitation. It opens up prospects for differentiating bostrycoidin and anhydrofusarubin. It should be noted that the exact position of the peak can be affected by pH and the dielectric properties of the medium and solvent, which can lead to some shifts in the absorption band, especially for anhydrofusarubin. However, in this case, shorter-wavelength (higher-energy) peaks can be used for differentiation.

## 4. Conclusions

For the first time, the geometry of the bostrycoidin molecule was modeled within the density functional theory. Optimization to the most stable minimum was performed, and the structural peculiarities were discussed. The possibility of conformational isomers and tautomers was studied. It was found that such a state is about 3 kcal/mol higher in energy. Compared with naphthazarin and anhydrofusarubin, tautomeric states for bostrycoidin have a significantly lower probability (about 1%). 

The vibrational spectrum for the bostrycoidin molecule (both IR absorption and Raman activity) is calculated. A comparison is made with the available experimental data in various studies, as well as with the spectra of structurally related anhydrofusarubin and naphthazarin. The description highlights the practically important features of the IR absorption spectra. The most active IR peaks are assigned. The presence of intense characteristic peaks at 1585 and 1632 cm^−1^ is noted, similar to the previously described maxima for anhydrofusarubin and naphthazarin. The most active IR peak is predicted to be at 1260 cm^−1^. The characteristic vibrational modes with peak frequencies of 1218, 1355, and 1362 cm^−1^ are obtained.

In addition, the calculation of the electron absorption spectra was performed in the 185–700 nm region. The results obtained are in good agreement with the available experimental data. The observed peaks were explained based on electron transitions. The longest-wavelength experimental band corresponded with a fairly high accuracy to the theoretically predicted peak at about 500 nm, attributed to the HOMO–LUMO transition, and its wavelength significantly differs from the longest-wavelength peak of a structurally similar anhydrofusarubin.

The theoretically studied structural features and optical properties of the bostrycoidin molecule can further serve as a foundation for the development of subsequent specific optical methods for the differentiation of this Fusarium pigment and detection of Fusarium infection in particular.

## Figures and Tables

**Table 1:** The selected bond lengths and angles for bostrycoidin molecule in GP. For comparison reasons, the corresponding bond lengths and angles of anhydrofusarubin [32] (see notations on Figure 1).

	Bond	Bond Length, Å	
		Bostrycoidin	Anhydrofusarubin
**Ring I**	C7C8	1.401	1.401
	C8C9	1.410	1.407
	C9C10	1.376	1.386
	C10C11	1.434	1.424
	C11C6	1.396	1.401
	C8O29	1.333	1.339
	O29H30	0.997	-
	C11O31	1.330	1.336
	O31H32	0.994	-
	C10O24	1.343	1.335
	O24C25	1.424	1.426
	C9H14	1.080	-
	C25H26	1.087	-
	C25H27	1.093	-
	C25H28	1.093	-
**Ring II**	C2C20	1.477	1.458
	C20C7	1.449	1.455
	C7C6	1.430	1.420
	C6C22	1.457	1.454
	C22C3	1.486	1.498
	C20O21	1.245	1.248
	C22O23	1.240	1.236
**Ring III**	N15C1	1.326	-
	C1C2	1.398	-
	C2C3	1.398	-
	C3C4	1.390	-
	C4C5	1.393	-
	C5N15	1.346	-
	C1H12	1.084	-
	C4H13	1.082	-
	C5C16	1.502	1.491
	C16H17	1.090	-
	C16H18	1.093	-
	C16H19	1.093	-
	Angle	Angle, °	
**Ring I**	C7C8C9	120.3	119.6
	C8C9C10	120.9	121.4
	C9C10C11	120.1	119.5
	C10C11C6	119.2	119.7
	C11C6C7	120.6	120.2
	C6C11O31	123.4	122.4
	C7C8O29	122.3	122.0
	C11O31H32	105.7	106.2
	C8O29H30	105.7	105.5
	C11C10O24	114.5	112.2
	C8C9H14	117.3	-
**Ring II**	C2C20C7	117.6	118.5
	C20C7C6	121.5	120.4
	C7C6C22	120.8	120.7
	C6C22C3	117.7	117.4
	C22C3C2	120.9	121.2
	C2C20O21	120.3	119.7
	C3C22O23	120.0	119.6
	C20C7C8	119.5	-
	C22C6C11	118.6	-
**Ring III**	N15C1C2	123.7	-
	C1C2C3	117.7	-
	C2C3C4	118.8	-
	C3C4C5	119.4	-
	C5N15C1	122.0	-
	C4C5C16	121.8	-
	C5C4H13	121.5	-
	N15C1H12	117.3	-

**Table 2:** UV–Vis absorbance peaks in chloroform solution.

Theory					Experiment	
					[2]	[19]
Peak №	Excited State	Orbitals with >10% Contribution (Percent)	Oscillator Strength	Wavelength, nm (Energy, eV)	Wavelength, nm	
1	1	74 -> 75 (99)	0.2196	498.43 (2.4875)	497 (475sh * and 525sh) *	492 (472sh * and 526sh *)
2	5	74 -> 76 (85)	0.1050	340.43 (3.6420)		
					
	and				320	324
				327.46		
	7	71 -> 75 (85)	0.804	(3.7863)		
3	11	73 -> 76 (85)	0.6683	256.73	251	252
				(4.8293)		
4	22	65 ->75 (37)	0.5449	205.74	N.O. **	204
		68 -> 76 (27)		(6.0263)		
		71 -> 77 (19)				

* probably the shoulders, a manifestation of the fine electron-vibrational structure, ** N.O.—not observed.

## Data Availability

The data presented in this study are available in the article.

## Supplementary Materials

The following supporting information can be downloaded at: https://www.mdpi.com/article/10.3390/molecules29194765/s1, Table S1: The selected bond lengths and angles for bostrycoidin and naphthazarin molecules in gas phase (GP). The optimization of naphthazarine molecule was performed in the same way as bostrycoidin; Figure S1: Potential energy scan with C11C10O24C25 dihedral angle change for the molecule in gas phase (blue) and chloroform solvent (red); Table S2: Conformational isomers and transition states found during changes of C11C10O24C25 dihedral angle in gas phase and chloroform (see Figure 2). In the case of transition states, the imaginary frequencies and atomic displacements in them are demonstrated. In the case of stable conformational isomers, the imaginary frequencies are absent. In order to demonstrate the enantiomeric forms the total and relative energies as well as imaginary frequencies are demonstrated with high precision. In our study we suppose that the agreement between relative energies of enantiomeric forms is within 0.1 kcal/mol and between imaginary frequencies is 0.1 cm^−1^; Table S3: Total and relative energies of tautomers and corresponding transition states during the transition of one hydrogen from the OH group to oxygen in O=C with the paths demonstrated at Figure 3 and Figure 4; Figure S2: Scaled theoretical Raman activity spectrum of bostrycoidin molecule in gas phase; Table S4: Interpretation of theoretically predicted vibrational modes in the 375–4000 cm^−1^ region for bostrycoidin molecule in gas phase (G.P.); Table S5: Comparison* of vibrational modes frequencies in gas phase and in CHCl3 solution. In the case of calculations in CHCl3 solution, the IR intensities and Raman activities are demonstrated; Figure S3: Difference of calculated frequency for corresponding vibrational modes in gas phase (ωG.P.) and in CHCl3 solution (ωCHCl3); Figure S4: Theoretical IR absorbance (a) and Raman activity (b) spectrum of bostrycoidin molecule in CHCl_3_ solution; Table S6: UV–Vis absorbance peaks in chloroform solution for tautomers; Figure S5: The UV–Vis spectrum in the 185–700 nm region for single bostrycoidin molecule in chloroform in most stable state 1 (a) and intermediate minimum states 8 (b), 9 (c), and 7 (d).
