# Supplementary material for: Investigation of Structural and Spectral Peculiarities of Fusarium sp. Indicator Pigment Bostrycoidin
Source: Molecules. 2024 Oct 8;29(19):4765. doi: 10.3390/molecules29194765 (PMC11478326; doi:10.3390/molecules29194765)
Supplement: Supplementary file 1 [file molecules-29-04765-s001.zip › molecules-3196307-supplementary.pdf]

Supporting information

Investigation of Structural and Spectral Peculiarities of *Fusarium* sp. Indicator Pigment Bostrycoidin

.

Anastasia Povolotckaia[1,2]*, Dmitrii Pankin[2], Vasiliy Novikov[1], Evgeniy Borisov[2], Sergey Kuznetsov[1], Alexey Dorokhov[1], Anatoliy Gulyaev[1], Elena Zavyalova[1,3], Rugiya Alieva[3], Sergey Akulov[1], Sergey Belousov[4], Maxim Moskovskiy[1]

[1] Federal Scientific Agro-Engineering Center VIM, 1st Institutskiy proezd 5, 109428 Moscow, Russia
[2] Center for Optical and Laser Materials Research, St. Petersburg State University, Ulianovskaya 5, 198504 St.Petersburg, Russia
[3] Chemistry Department, Lomonosov Moscow State University, 119991 Moscow, Russia
[4] Department of Processes and Machines in Agribusiness, Kuban State Agrarian University Named after I.T. Trubilin, 350044 Krasnodar, Russia
***** Correspondence: anastasia.povolotckaia@spbu.ru;

Table S1. The selected bond lengths and angles for bostrycoidin and naphthazarin molecules in gas phase (GP). The optimization of naphthazarine molecule was performed in the same way as bostrycoidin.

Bostrycoidin			Napthtazarine
	Bond	Bond length, A	Bond length, A
Ring I	C7C8	1.401	1.401
	C8C9	1.410	1.414
	C9C10	1.376	1.369
	C10C11	1.434	1.414
	C11C6	1.396	1.401
	C8O29	1.333	1.334
	O29H30	0.997	0.993
	C11O31	1.330	1.334
	O31H32	0.994	0.992
	H30O21	1.643	1.683
	H32O23	1.651	1.683
Ring II	C2C20	1.477	1.473
	C20C7	1.449	1.457
	C7C6	1.430	1.422
	C6C22	1.457	1.457
	C22C3	1.486	1.473
	C20O21	1.245	1.241
	C22O23	1.240	1.241
	Angle	Angle, ^{o}	Angle, ^{o}
Ring I	C7C8C9	120.3	119.2
	C8C9C10	120.9	120.9

	C9C10C11	120.1	120.9
	C10C11C6	119.2	119.2
	C11C6C7	120.6	119.9
	C6C11O31	123.4	122.6
	C7C8O29	122.3	122.6
	C11O31H32	105.7	106.3
	C8O29H30	105.7	106.3
Ring II	C2C20C7	117.6	117.8
	C20C7C6	121.5	120.3
	C7C6C22	120.8	120.4
	C6C22C3	117.7	117.8
	C22C3C2	120.9	121.9
	C2C20O21	120.3	119.8
	C3C22O23	120.0	119.8
	C20C7C8	119.5	119.7
	C22C6C11	118.6	119.7

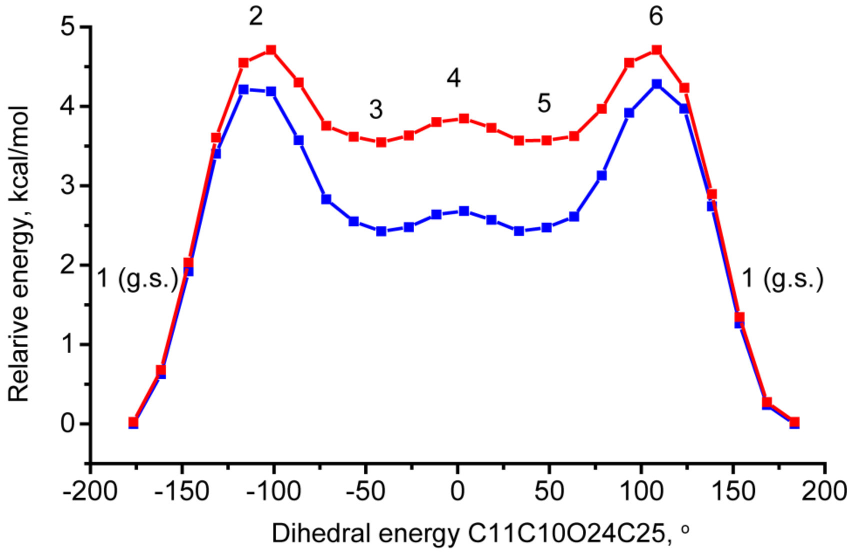

Figure S1. Potential energy scan with C11C10O24C25 dihedral angle change for the molecule in gas phase (blue) and chloroform solvent (red).

Table S2. Conformational isomers and transition states found during changes of C11C10O24C25 dihedral angle in gas phase and chloroform (see Figure 2). In the case of transition states the imaginary frequencies and atomic displacements in them are demonstrated. In the case of stable conformational isomers, the imaginary frequencies are absent. In order to demonstrate the enantiomeric forms the total and relative energies as well as imaginary frequencies are demonstrated with high precision. In our study we suppose that the agreement between relative energies of enantiomeric forms is within 0.1 kcal/mol and between imaginary frequencies is 0.1 cm^{-1}.

Type	State (optionally comments)	Total energy in Ha (relative energy in kcal/mol) in gas phase	Total energy in Ha (relative energy in kcal/mol) in chloroform
Stable conformational isomers	1 (ground states, Cs symmetry) 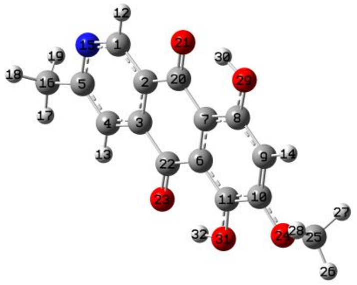	-1009.404117 (0)	-1009.411731 (0)
	3 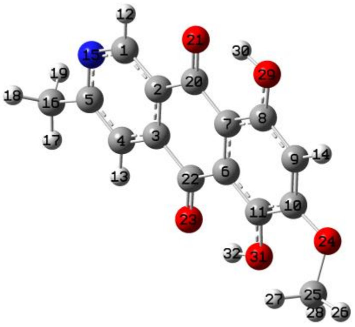	-1009.400269 (2.41)	-1009.406095 (3.54)
	5 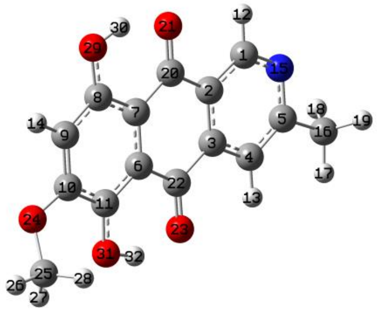	-1009.400269 (2.41)	-1009.406095 (3.54)
Transition state	2 (1 imaginary frequency) 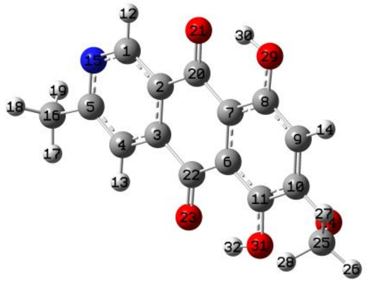	-1009.397282 (4.29) (imaginary frequency 77.06i cm^{-1}) 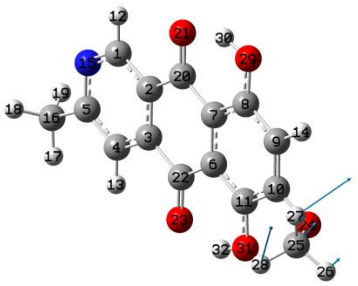	-1009.404198 (4.73) (imaginary frequency 75.88i cm^{-1}) 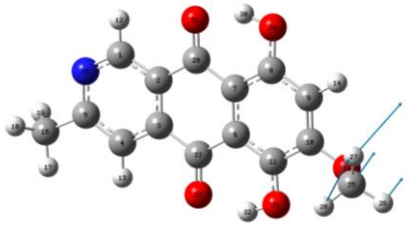
	4 (1 imaginary frequency, Cs symmetry)	-1009,39985 (2.68) (imaginary frequency 47.81i cm^{-1})	-1009.405616 (3.84) (imaginary frequency 40.78i cm^{-1})

	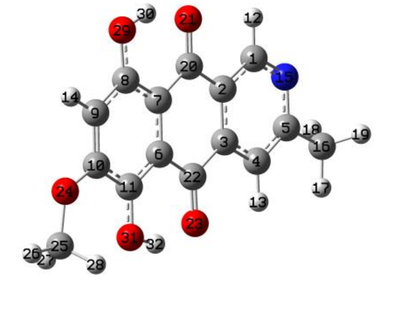	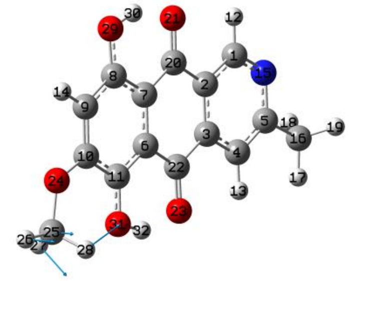	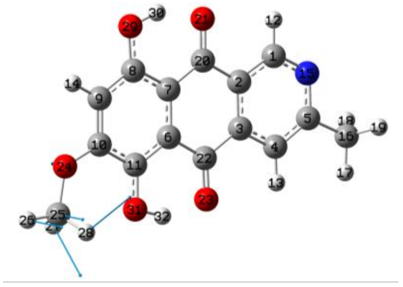
	6 (1 imaginary frequency) 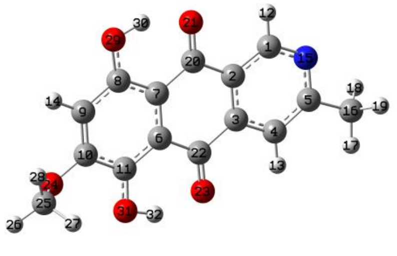	-1009.397282 (4.29) (imaginary frequency 77.03i cm^{-1}) 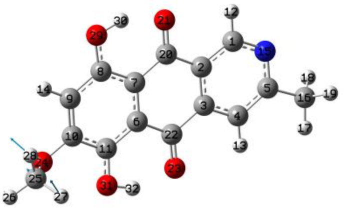	-1009.404198 (4.73) (imaginary frequency 75.91i cm^{-1}) 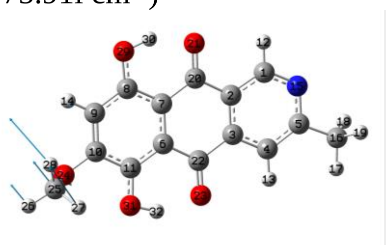

Table S3. Total and relative energies of tautomers and corresponding transition states during the transition of one hydrogen from the OH group to oxygen in O=C with the paths demonstrated at figure 3 and 4.

	State (optionally comments)	Total energy in Ha (relative energy in kcal/mol) in gas phase (in case of transition states the atomic displacements and imaginary frequency is demonstrated)	Total energy in Ha (relative energy in kcal/mol) in chloroform (in case of transition states the atomic displacements and imaginary frequency is demonstrated)
	1 (ground states, Cs symmetry) 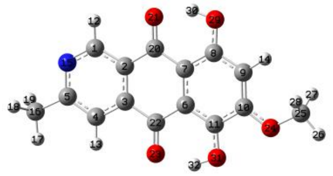	-1009.404117 (0)	-1009.411731 (0)
Path 1	Transition state 1->7 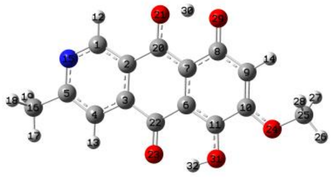	-1009.396016 (5.08) (imaginary frequency 896.05i cm^{-1}) 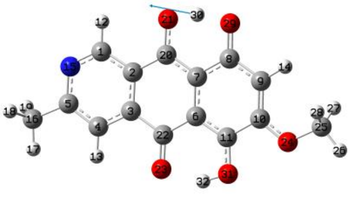	-1009.403871 (4.93) (imaginary frequency 891.81i cm^{-1}) 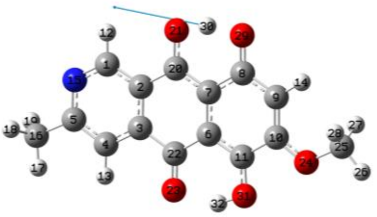
	7	-1009.396830 (4.57)	-1009.404651 (4.44)

	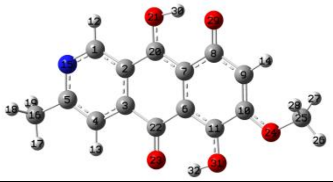		
	Transition state 7->8<br>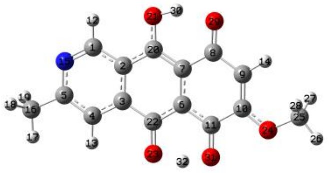	-1009.393367 (6.75)<br>(imaginary frequency 1190.31i cm^{-1})<br>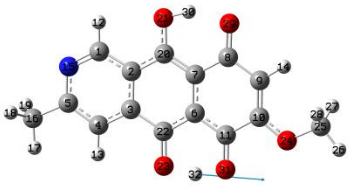	-1009.401251 (6.58)<br>(imaginary frequency 1186.15i cm^{-1})<br>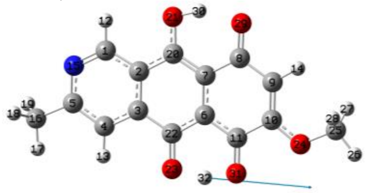
	8<br>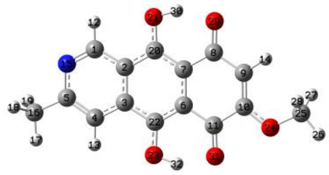	-1009.399393 (2.96)	-1009.407217 (2.83)
Path 2	Transition state 1->9<br>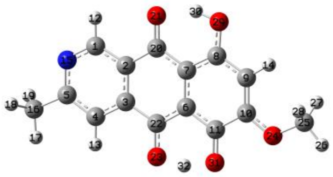	-1009.395542 (5.38)<br>(imaginary frequency 1017.54i cm^{-1})<br>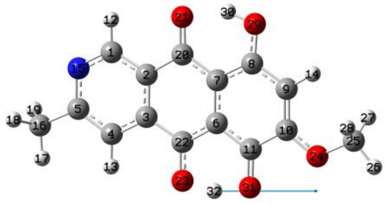	-1009.403551 (5.13)<br>(imaginary frequency 1035.25i cm^{-1})<br>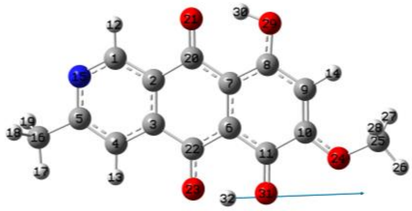
	9<br>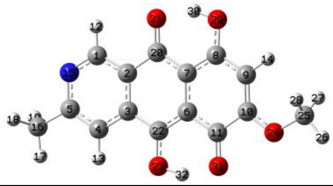	-1009.396977 (4.48)	-1009.40509652 (4.16)
	Transition state 9->8<br>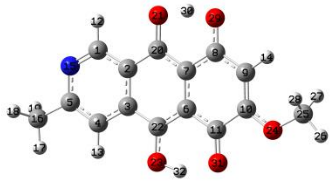	-1009.394155 (6.25)<br>(imaginary frequency 1131.12i cm^{-1})<br>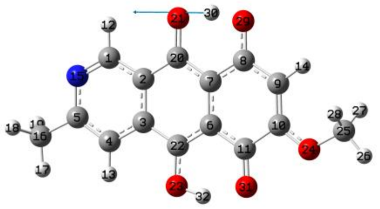	-1009.402305 (5.91)<br>(imaginary frequency 1127.26i cm^{-1})<br>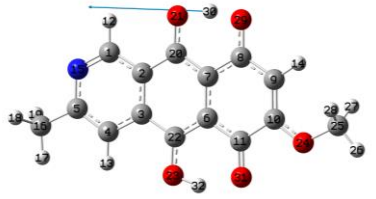

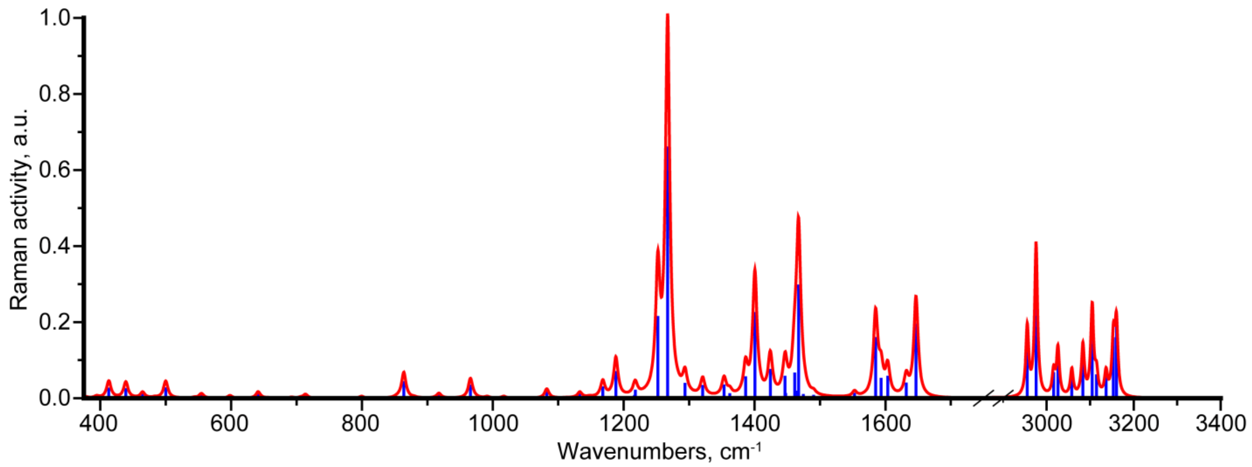

Figure S2. Scaled theoretical Raman activity spectrum of bostrycoidin molecule in gas phase.

Table S4. Interpretation of theoretically predicted vibrational modes in the 375-4000 cm^{-1} region for bostrycoidin molecule in gas phase (G.P.).

Mode №	Frequency, cm^{-1}	Scaled frequency, cm^{-1}	IR intensities (KM/Mole)	Raman scattering activities (A^4/AMU)	Assignment
17	385.01	377.3098	1.2493	2.0719	δ(C16C5C4), δ(C1C2C20), δ(C4C3C22), δ(C20C7C8), δ(C10O24C25)
18	402.65	394.597	4.7028	2.9402	predominantly δ(C16C5C4)
19	421.48	413.0504	14.9718	27.264	predominantly δ(O29C8C7), δ(O21C20C7), δ(O31C11C6), δ(O23C22C6), with smaller contribution δ(C4C3C22), δ(C16C5N15)
20	422.49	414.0402	0	0.6803	tors(H32O31C11C6), tors(H30O29C8C7), tors(C8C7C6C11), tors(C8C9C10C11), tors(C8C9O24C25)
21	435.92	427.2016	5.7432	0.0506	predominantly tors(C5N15C1H12), tors(N15C5C4H13) , tors(C1C2C3C4)
22	448.05	439.089	8.4261	26.3559	δ(C2C20C7), δ(C3C22C6), δ(C7C8C9), δ(C6C11C10), δ(C10O24C25)
23	474.17	464.6866	4.4976	9.4117	δ(C7C8C9), δ(C6C11C10), δ(C10O24C25)
24	493.24	483.3752	1.5264	1.2562	ρ(H18C16H19), tors(C5N15C1H12), tors(C1C2C3C4), tors(N15C5C4H13) , tors(C20C7C6C22), tors (H14C9C10C11)
25	510.24	500.0352	4.5327	28.4794	predominantly δ(N15C5C4), δ(C1C2C3), δ(C2C20C7), δ(C3C22C6), δ(O29C8C7), δ(C10O24C25)
26	555.2	544.096	0.9223	1.1288	ρ(H18C16H19), tors(H14C9C10C11),

					tors(C5N15C1H12), tors(C1C2C3C4), tors(C20C7C6C22)
27	565.91	554.5918	6.1774	7.7201	δ(N15C5C16), δ(N15C5C4), δ(C2C3C22), δ(C2C20C7), δ(C6C11O31), δ(C10O2C25) δ(C8C9C10)
28	610.47	598.2606	25.0459	4.4676	predominantly δ(C8C9C10), δ(C9C10O24), δ(C10C11O31), δ(C7C8O29), δ(C7C20O21), δ(C1C2C20), δ(C16C5C4)
29	654.41	641.3218	3.5483	11.2067	predominantly δ(C5C4C3), δ(N15C5C4), δ(C7C6C22), δ(O29C8C7) δ(C9C10C11)
30	670.63	657.2174	2.1834	0.6543	ρ(H18C16H19), tors(N15C5C4H13), tors(C20C7C6C22), tors(H30O29C8C9), tors(H14C9C10C11)
31	706.48	692.3504	4.4844	1.794	δ(C1N15C5), δ(C3C22O23), δ(C2C20O21), δ(C4C3C22), δ(C6C7C11)
32	720.88	706.4624	1.1217	1.2096	ρ(H18C16H19), tors(C5N15C1H12), tors(N15C5C4H13) , tors(C20C7C6C22), tors (H14C9C10C11)
33	728.2	713.636	22.1453	6.6887	predominantly δ(C10O24C25), δ(O31C11C10), δ(O29C8C9), δ(N15C1C2), δ(C5C4C3), δ(C3C22C6)
34	752.32	737.2736	0.4796	0.1034	tors(C5N15C1H12), tors(C1C2C3C4), tors(H30O29C8C7), tors(H32O31C11C10), tors (H14C9C10C11)
35	773.14	757,6772	1.6642	0.3549	tors(C5N15C1H12), tors(C20C7C6C22), tors(H30O29C8C7), tors(H32O31C11C10)
36	815.66	799.3468	9.7208	3.3115	δ(C1C2C3), δ(O23C22C6), δ(N15C5C4), δ(O29C8C9)
37	830.07	813.4686	0.0178	0.1589	tors(H32O31C11C6), tors(N15C5C4H13), tors(C5N15C1H12), tors(C20C2C3C22), tors(C8C7C6C11), tors(H30O29C8C7), tors(H14C9C10C11)
38	870.25	852.845	122.6296	1.2966	predominantly tors(H32O31C11C6)
39	874.2	856.716	8.4519	0.9867	predominantly tors(H32O31C11C6), tors(H142C9C10C11)
40	881.55	863.919	1.2084	46.9063	δ(C7C8C9), δ(O21C20C7), δ(O31C11C6), δ(C3C22C6), v(C3C22),

					ν(C5C16)
41	897.49	879.5402	61.6139	1.31	predominantly tors(H30O29C8C7)
42	930.03	911.4294	3.5569	0.3367	predominantly tors(N15C5C4H13) with smaller contribution ρ(H18C16H19)
43	936.34	917.6132	39.7203	8.7424	predominantly δ(N15C5C4), ν(C3C22), ν(C22C6), ν(C2C20), δ(C6C7C8), δ(C7C20O21), ρ($C16H_3$)
44	985.55	965.839	52.906	36.7759	ν(C8O29), ν(C8C7), ν(C8C9), ν(O24C25), δ(C9C10O24), δ(N15C1H12), δ(C5C4H13), δ(C22C6H11), δ(C20C2C 1)
45	992.31	972.4638	0.4591	0.9137	tors(C5N15C1H12)
46	1011.6	991.368	25.9964	2.6323	predominantly ρ($C16H_3$), ν(O24C25),
47	1037.37	1016.6226	9.4581	3.3894	predominantly ρ($C16H_3$), ν(O24C25), δ(C5C4H13), δ(C10C9H14)
48	1066.46	1045.1308	6.2841	0.7455	ρ($C16H_3$), τ(H19C16H18), with smaller addition tors(N15C5C4H13), tors(C5N15C1H12)
49	1104.57	1082.4786	0.6946	17.6444	predominantly ν(C3C22), δ(N15C1H12), δ(C5C4H13), ν(C1C2), ν(C22C6), ν(O24C25), δ(C11C10C24),
50	1156.04	1132.9192	77.0389	10.5789	predominantly ν(C2C20), ν(C16C5), ν(C4C3),ν(C20C7), ν(C3C22), ν(C7C8), ν(C8C9), δ(C7C8C9), δ(C4C3C2), δ(C2C20C7), δ(O24C25H26), δ(C8O29H30), δ(C11O31H32)
51	1171.35	1147.923	1.2168	1.667	ρ($C25H_3$), τ(H27C25H28)
52	1191.89	1168.0522	27.7099	31.1324	ρ($C25H_3$), δ(C5C4H13), δ(C10C9H14), δ(N15C1H12)
53	1212.32	1188.0736	35.5254	77.0161	δ(C10C9H14), δ(C5C4H13), ρ($C25H_3$)
54	1241.41	1216.5818	50.1766	5.3097	δ(C10C9H14), δ(C5C4H13)
55	1242.64	1217.7872	184.5003	21.0619	ν(O24 C10), δ(C10C9H14), ρ($C25H_3$), δ(O24C25H26)
56	1277.81	1252.2538	28.9141	242.7245	predominantly ν_{sym}(N15C1C2) and ν_{sym}(C5C4C3) like in ν19b in benzene ring)
57	1292.97	1267.1106	565.5788	752.4838	δ(N15C1H12), δ(C5C4H13), δ(C8O29H30), ν(C10O24), anti-phase ν19b benzene ring mode-like in adjacent rings of polycyclic part (ν_{sym}(C6C11C10) and

					ν_{sym}(C7C8C9) like in v19b in benzene ring; ν_{sym}(C2C20C7) and ν_{sym}(C3C22C6) like in v19b in benzene ring; ν_{sym}(N15C1C2) and ν_{sym}(C5C4C3) like in v19b in benzene ring)
58	1309.59	1283.3982	6.6982	0.9051	predominantly δ(N15C1H12), δ(C5C4H13)
59	1320.05	1293.649	276.8592	41.5904	predominantly ν(C7C6), ν(O31C11), ν(C8CO29), ν(O24C10), δ(C10C9H14), δ(C8O29H30) with smaller contribution of ν(N15C1), ν(C5C4)
60	1347.72	1320.7656	138.5316	35.2769	predominantly ν(O31C11), (ν_{sym}(C6C11C10) and ν_{sym}(C7C8C9) like in v19a in benzene ring), ν(C22C6), δ(C8O29H30)
61	1381.06	1353.4388	322.6785	37.0825	predominantly δ(C11O31H32), δ(C8O29H30), ν(C7C8) with smaller addition (ν_{sym}(N15C1C2) and ν_{sym}(C5C4C3) like in v19a in benzene ring), (ν_{sym}(C2C20C7) and ν_{sym}(C3C22C6) like in v19a in benzene ring), ν_{asym} ($C16H_3$)
62	1389.9	1362.102	228.5395	10.8064	predominantly δ(C11O31H32), ν(C20C7), ν(C22C6), ν_{asym}(C6C11C10), with smaller addition δ(C10C9H14), δ(C8O29H30), ν_{sym} ($C25H_3$), ν_{asym} ($C16H_3$), δ(N15C1H12), δ(C5C4H13)
63	1414.64	1386.3472	24.3607	61.4198	ν_{sym} ($C16H_3$), ν(C16C5)
64	1429.07	1400.4886	25.8362	255.095	(ν_{sym}(N15C1C2) and ν_{sym}(C5C4C3) like in v19a in benzene ring), ν_{asym}(C3C22C6), ν_{sym}(C20C7C6), ν(C8O29), ν(C9C10), ν_{sym} ($C25H_3$), ν_{asym} ($C16H_3$)
65	1453.17	1424.1066	140.0124	83.8866	predominantly ν_{sym} ($C25H_3$), δ(C8O29H30), δ(C11O31H32), ν(O29C8), ν(C7C6), ν(C20C7), ν(C9C10), ν(C22C6), ν_{asym} ($C16H_3$)
66	1476.03	1446.5094	246.8104	63.0904	ν_{sym} ($C25H_3$), ν(O29C8), δ(C10C9H14), δ(C8O29H30), δ(C8O29H30), δ(C11O31H32)
67	1477.06	1447.5188	7.2388	11.173	predominantly ν_{asym} ($C16H_3$)
68	1491.1	1461.278	30.3102	72.8939	predominantly

					v_{asym} ($C16H_3$)
69	1494.9	1465.002	9.4801	18.3824	predominantly<br>v_{asym} ($C25H_3$)
70	1497.3	1467.354	129.7047	337.4483	predominantly<br>v_{sym} ($C25H_3$)<br>with smaller addition of<br>v(C7C8), v(C11C10), v(O24 C10), δ(C8O29H30), δ_{asym}($C16H_3$)
71	1504.63	1474.5374	22.3186	8.3057	predominantly<br>v_{asym} ($C25H_3$)
72	1507.53	1477.3794	17.4348	1.2568	predominantly<br>δ(N15C1H12), δ(C5C4H13)<br>with smaller addition of<br>v(C1C2), v(C2C3), v(C4C3) , v(N15C5), δ_{asym}($C16H_3$), δ_{asym}($C25H_3$)
73	1520.82	1490.4036	248.344	5.7553	predominantly<br>δ(C11O31H32), δ(C8O29H30), δ_{sym}($C25H_3$), v(C8C9), v(C9C10), v(C10C11), v(C11C6), v(C20O21), δ(C10C9H14)
74	1584.5	1552.81	8.2097	11.4068	predominantly<br>(v_{sym}(N15C1C2) and v_{sym}(C5C4C3) like in v8a in benzene ring),<br>δ(C8O29H30), δ(C11O31H32),<br>δ_{asym}($C16H_3$), δ(C3C4H13), δ(C2C1H12)
75	1617.41	1585.0618	321.4938	179.484	predominantly<br>v(O21C20), v(O23C22),<br>(v_{asym}(N15C1C2) and v_{asym}(C5C4C3) like in 8b benzene ring),<br>v(C7C8), v(C6C11), δ(N15C1H12), δ(C5C4H13), δ(C11O31H32)
76	1625.9	1593.382	65.0382	56.7543	predominantly<br>δ(C8O29H30), δ(C11O31H32), v(C9C8), δ(C10C9H14), v(C7C8), v(C6C11)
77	1636.22	1603.4956	29.0139	63.1827	predominantly<br>δ(C8O29H30), δ(C11O31H32),<br>(v_{asym}(C7C8C9) and v_{asym}(C6C11C10) like in v8b in benzene ring),<br>δ(C10C9H14)
78	1665.17	1631.8666	292.8196	42.4276	v(O21C20), v(O23C22), δ(C8O29H30), (v_{asym}(N15C1C2) and v_{asym}(C5C4C3) like in 8b benzene ring), v(C7C8), δ(N15C1H12), δ(C5C4H13), δ(C11O31H32), δ(C10C9H14)
79	1680.35	1646.743	24.607	219.4296	v(O23C22), v(O21C20), v(C2C3), v(C7C6), v(C9C10), δ(C8O29H30), δ(C11O31H32), δ(C10C9H14)
80	3015.83	2955.5134	51.858	209.1398	v_{sym}($C25H_3$)
81	3036.49	2975.7602	12.5113	464.7585	v_{sym}($C18H_3$)
82	3077.71	3016.1558	26.4446	73.7718	v_{asym}(H28C25H27)

83	3087.78	3026.0244	7.9776	147.0532	v_{asym}(H19C18H16)
84	3120.28	3057.8744	13.0904	83.4062	v_{asym}(C18H$_3$)
85	3146.33	3083.4034	16.8267	159.6789	v_{asym}(C25H$_3$)
86	3168.05	3104.689	158.7212	282.7758	v(H30O29)
87	3178.21	3114.6458	4.351	67.2091	v(H12C1)
88	3200.52	3136.5096	3.3399	73.727	v(H13C4)
89	3217.81	3153.4538	9.5825	178.8642	v(H14C9)
90	3224.91	3160.4118	117.5487	226.6496	v(H32O31)

The notations v, δ, w, ρ, τ and tors corresponds to the stretching, bending, wagging, rocking, twisting and torsional modes. The sym and asym corresponds to the symmetric and antisymmetric vibrations. In case of v_{sym}(XYZ) and v_{asym}(XYZ) (here X,Y,Z are the atom labels with their number, e.g. v_{asym}(H28C25H27)) the symmetric and antisymmetric stretching water-like vibrations are supposed.

Table S5. Comparison* of vibrational modes frequencies in gas phase and in $CHCl_3$ solution. In the case of calculations in CHCl3 solution the IR intensities and Raman activities are demonstrated.

Mode №	Frequency in gas phase, cm^{-1}	Scaled frequency in gas phase, cm^{-1}	Frequency in $CHCl_3$, cm^{-1}	Scaled frequency in $CHCl_3$,cm^{-1}	IR intensities in CHCl3 (KM/Mole)	Raman scattering activities $CHCl_3$ (A^4/AMU)	Frequency difference (frequency for the gas phase case minus frequency for the $CHCl_3$ solution), cm^{-1}
17	385.01	377.3098	385.6	377.888	2.4911	5.9547	-0.59
18	402.65	394.597	403.87	395.7926	10.0249	3.0832	-1.22
19	421.48	413.0504	421.21	412.7858	23.9421	66.4795	0.27
20	422.49	414.0402	423.41	414.9418	8E-4	1.7953	-0.92
21	435.92	427.2016	435.68	426.9664	7.5705	0.0504	0.24
22	448.05	439.089	447.41	438.4618	13.3569	84.697	0.64
23	474.17	464.6866	472.72	463.2656	9.6734	26.1918	1.45
24	493.24	483.3752	492.99	483.1302	2.1789	2.915	0.25
25	510.24	500.0352	509.87	499.6726	8.5111	66.7554	0.37
26	555.2	544.096	555.65	544.537	1.4014	2.6569	-0.45
27	565.91	554.5918	565.97	554.6506	10.0661	23.9187	-0.06
28	610.47	598.2606	609.36	597.1728	46.5567	16.3092	1.11
29	654.41	641.3218	653.53	640.4594	7.2154	31.5747	0.88
30	670.63	657.2174	671.09	657.6682	3.1759	1.8366	-0.46
31	706.48	692.3504	706.62	692.4876	5.5364	3.0111	-0.14
32	720.88	706.4624	721.16	706.7368	1.551	2.5017	-0.28
33	728.2	713.636	727.08	712.5384	36.7334	13.6616	1.12
34	752.32	737.2736	751.62	736.5876	0.8653	0.1689	0.7
35	773.14	757.6772	772.17	756.7266	1.5582	0.5768	0.97

36	815.66	799.3468	814.59	798.2982	19.3999	8.6996	1.07
37	830.07	813.4686	828.38	811.8124	0.9316	0.4996	1.69
38	870.25	852.845	853.78	836.7044	135.7681	1.9753	16.47
39	874.2	856.716	869.7	852.306	99.4659	1.2435	4.5
40	881.55	863.919	880.9	863.282	3.2114	102.1087	0.65
41	897.49	879.5402	878.85	861.273	21.8112	2.5828	18.64
42	930.03	911.4294	929.67	911.0766	4.6195	0.9984	0.36
43	936.34	917.6132	935.84	917.1232	74.7676	15.877	0.5
44	985.55	965.839	981.72	962.0856	98.0511	127.6538	3.83
45	992.31	972.4638	989.76	969.9648	0.4329	1.4216	2.55
46	1011.6	991.368	1008.7	988.526	43.4937	9.4419	2.9
47	1037.37	1016.6226	1032.8	1012.144	8.6043	2.5784	4.57
48	1066.46	1045.1308	1065.18	1043.8764	9.122	2.2521	1.28
49	1104.57	1082.4786	1102.47	1080.4206	2.4509	48.4684	2.1
50	1156.04	1132.9192	1155.42	1132.3116	150.2404	26.9321	0.62
51	1171.35	1147.923	1170.08	1146.6784	1.7788	2.0469	1.27
52	1191.89	1168.0522	1188.62	1164.,8476	37.44	86.2551	3.27
53	1212.32	1188.0736	1210.14	1185.9372	85.3247	191.7462	2.18
54	1241.41	1216.5818	1241.43	1216.6014	68.1872	34.7353	-0.02
55	1242.64	1217.7872	1235.74	1211.0252	324.6861	44.2241	6.9
56	1277.81	1252.2538	1276.89	1251.3522	2.739	482.9854	0.92
57	1292.97	1267.1106	1287.79	1262.0342	1226.9198	1965.4105	5.18
58	1309.59	1283.3982	1310.8	1284.584	198.2508	217.6855	-1.21
59	1320.05	1293.649	1307.87	1281.7126	223.3089	260.5007	12.18
60	1347.72	1320.7656	1344.43	1317.5414	216.4595	45.3125	3.29
61	1381.06	1353.4388	1374.65	1347.157	518.1185	156.4226	6.41
62	1389.9	1362.102	1387.94	1360.1812	364.5051	72.5463	1.96
63	1414.64	1386.3472	1411.33	1383.1034	32.1616	124.395	3.31
64	1429.07	1400.4886	1424.84	1396.3432	44.8661	823.9336	4.23
65	1453.17	1424.1066	1449.52	1420.5296	234.39	145.5423	3.65
66	1476.03	1446.5094	1466.6	1437.268	401.8062	492.1299	9.43
67	1477.06	1447.5188	1471.07	1441.6486	9.9239	16.7396	5.99
68	1491.1	1461.278	1486.04	1456.3192	50.7017	102.1114	5.06
69	1494.9	1465.002	1489.2	1459.416	12.6766	25.4171	5.7
70	1497.3	1467.354	1492.82	1462.9636	108.5524	963.244	4.48
71	1504.63	1474.5374	1498.76	1468.7848	27.098	9.9404	5.87
72	1507.53	1477.3794	1508.33	1478.1634	18.3471	46.6349	-0.8
73	1520.82	1490.4036	1515.57	1485.2586	343.619	22.6676	5.25
74	1584.5	1552.81	1582.09	1550.4482	19.57	30.8619	2.41
75	1617.41	1585.0618	1612.3	1580.054	571.1989	438.8822	5.11
76	1625.9	1593.382	1618.19	1585.8262	78.996	231.0539	7.71
77	1636.22	1603.4956	1632.58	1599.9284	63.991	261.2075	3.64
78	1665.17	1631.8666	1655.14	1622.0372	412.0107	145.3206	10.03
79	1680.35	1646.743	1673.12	1639.6576	43.2645	623.1275	7.23
80	3015.83	2955.5134	3025.83	2965.3134	60.0981	319.2043	-10
81	3036.49	2975.7602	3035.81	2975.0938	15.9869	791.5266	0.68
82	3077.71	3016.1558	3092.67	3030.8166	27.5324	120.3024	-14.96
83	3087.78	3026.0244	3087.08	3025.3384	10.8894	240.3623	0.7
84	3120.28	3057.8744	3121.91	3059.4718	18.0914	145.1333	-1.63
85	3146.33	3083.4034	3153.92	3090.8416	153.361	502.6992	-7.59

86	3168.05	3104.689	3154.37	3091.2826	154.1498	389.7611	13.68
87	3178.21	3114.6458	3177.23	3113.6854	6.7327	161.5893	0.98
88	3200.52	3136.5096	3204.23	3140.1454	1.0064	203.8681	-3.71
89	3217.81	3153.4538	3220.02	3155.6196	2.2016	223.4094	-2.21
90	3224.91	3160.4118	3207.3	3143.154	219.5144	698.6796	17.61

*the correspondence between equivalent vibrational modes in the gas phase and in solution was established on the basis of atomic displacements.

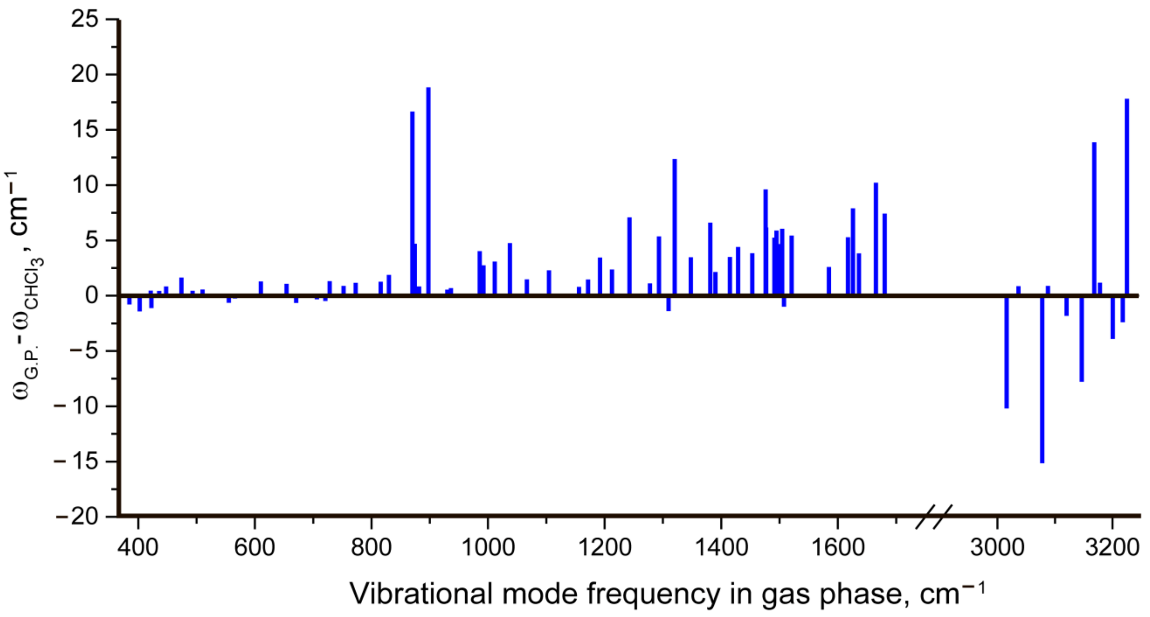

Figure S3. Difference of calculated frequency for corresponding vibrational modes in the gas phase ($\omega_{G.P.}$) and in $CHCl_3$ solution (ω_{CHCl3}).

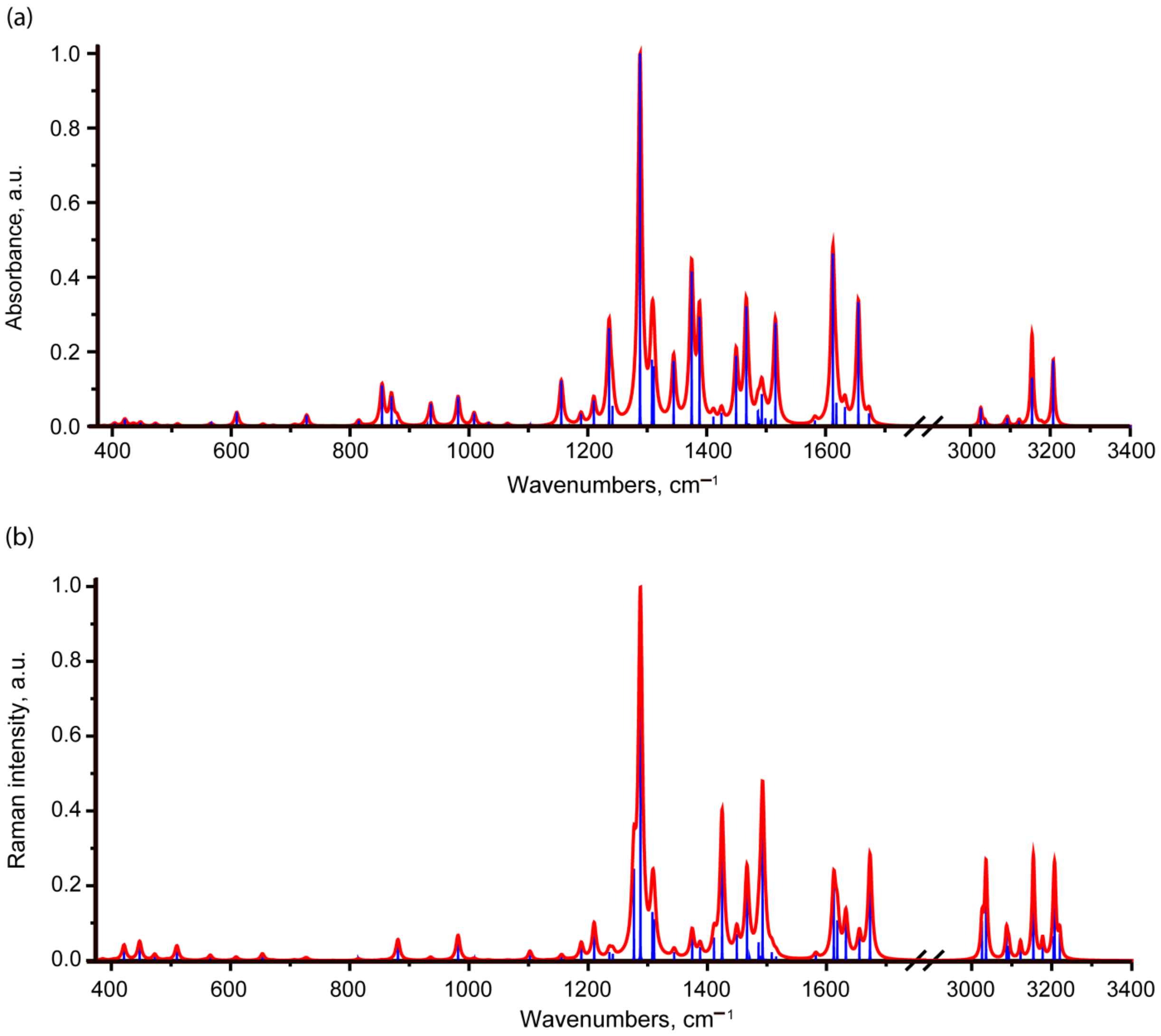

Figure S4. Theoretical IR absorbance (a) and Raman activity (b) spectrum of bostrycoidin molecule in $CHCl_3$ solution.

Table S6. UV−Vis absorbance peaks in chloroform solution for tautomers.

Intermediate minimum state number*	Wavelengths (energies) of transitions mainly forming a peak in calculated spectrum, nm (eV)			
	1	2	3	4
7	513.54 (2.4143)	354.45 (3.4979)<br>324.31 (3.8230)	263.06 (4.7131)	208.29 (5.9526)
8	516.11 (2.4023)	320.28 (3.8711)<br>362.56 (3.4197)<br>384.43 (3.2251)	268.89 (4.6109)	236.05 (5.2525)
9	533.43 (2.3243)	356.70 (3.4759)<br>335.18 (3.6990)	255.26 (4.8572)<br>252.30 (4.9141)	209.13 (5.9286)

* the numbering of intermediate minimum states is given in the figures 3 and 4 in the text

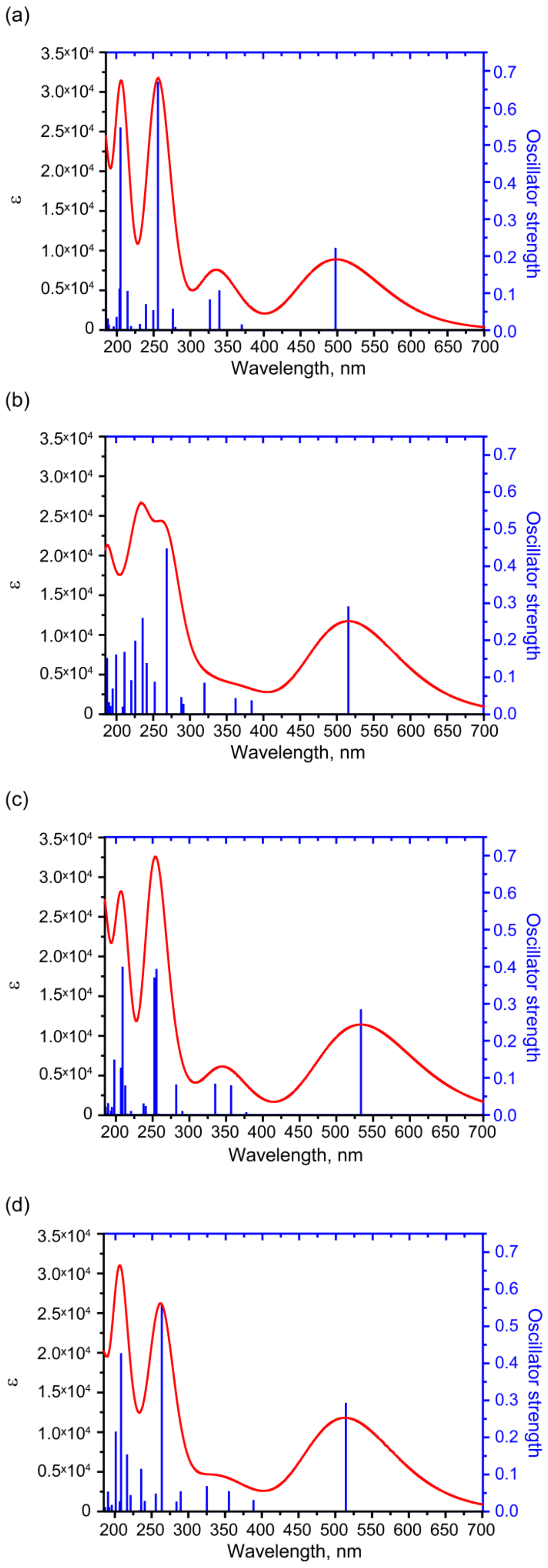

Figure S5. The UV–Vis spectrum in the 185-700 nm region for single bostrycoidin molecule in chloroform. in most stable state 1(a) and intermediate minimum states 8 (b), 9 (c) and 7 (d).

Comments on the spectra of tautomers.

For tautomer 8, the calculated UV-vis absorption spectrum differs quite significantly from the spectrum predicted for the most stable state. This is manifested in the low resolution of the two most intense absorption peaks 3 and 4 (see Table S5) as a result of a significant redshift of peak 4 (see Figure S5). For tautomers 7 and 9, the absorption spectra contain 3 and 4 clearly defined peaks, respectively, which is closer to the spectrum of the most stable state. For the given tautomers, the peak 4 corresponding to the HOMO-LUMO transition is shifted to the region of longer wavelengths, which indicates a higher stability and lower reactivity [1] for the found most stable state. In general, this is consistent with the closer calculated peak wavelengths as compared to corresponding experimental ones for the most stable state. The calculation does not predict transitions with a significant oscillator strength near 472 nm. At the same time, for tautomer 9 the longest wavelength peak is predicted at about 533 nm with other peaks being relatively close to those in the most stable state. The calculated peak at 533 nm for tautomer 9 may be close to the peak shoulder at 526 nm in the experimental spectrum (see table 2), however the smallest difference in HOMO-LUMO energies suggests at the same time much lower stability.

References

[1] Pankin, D.; Povolotckaia, A.; Borisov, E.; Belyakov, M.; Borzenko, S.; Gulyaev, A.; Moskovskiy, M. Theoretical Modelling of Structure, Vibrational and UV–Vis Absorbance Spectra of Rubrofusarin Molecule. Spectrochim Acta A Mol Biomol Spectrosc 2023, 293, 122469, doi:10.1016/j.saa.2023.122469
